# The Therapeutic Use of Impella Device in Cardiogenic Shock: A Systematic Review

**DOI:** 10.7759/cureus.30045

**Published:** 2022-10-07

**Authors:** Carlos Munoz Tello, Dawood Jamil, Hadrian Hoang-Vu Tran, Mafaz Mansoor, Samia Rauf Butt, Travis Satnarine, Pranuthi Ratna, Aditi Sarker, Adarsh Srinivas Ramesh, Lubna Mohammed

**Affiliations:** 1 General Medicine, Universidad Católica de Cuenca, Cuenca, ECU; 2 Internal Medicine, California Institute of Behavioral Neurosciences and Psychology, Fairfield, USA; 3 Medicine, California Institute of Behavioral Neurosciences and Psychology, Fairfield, USA; 4 General Practice, California Institute of Behavioral Neurosciences and Psychology, Fairfield, USA; 5 Pediatrics, Jackson Memorial Hospital, Miami, USA; 6 Medicine, Kamineni Academy of Medical Sciences and Research Centre (KAMSRC), Hyderabad, IND

**Keywords:** cardiovascular intervention, heart assist devices, heart failure, impella device, cardiogenic shock

## Abstract

Impella (Abiomed, Danvers, MA) devices nowadays have been linked to cardiogenic shock (CS) due to the importance of their use as therapeutic instruments. This study aims to review pathophysiologic mechanisms of cardiogenic shock and the implementation of Impella to overcome this condition. To investigate several different types of studies and analyze the use of Impella device in cardiogenic shock and the outcomes of heart malfunctioning and determine its positive and negative impacts as a therapeutic tool in cardiac ischemia and use as a resource in critical patients, we conducted a systematic review through different databases (PubMed, ScienceDirect, and Google Scholar) following the Preferred Reporting Items for Systematic Reviews and Meta-Analyses (PRISMA) checklist and used the Medical Subjects Heading (MeSH) search strategy to obtain significant articles. We found 883 papers in total, and after removing duplicates, applying inclusion/exclusion criteria, and finding the most significant information, we ended up with 30 articles that were reviewed containing information about the impact of Impella device in cardiogenic shock in different locations. The study strongly concludes that Impella device in the setting of cardiogenic shock has more advantages than disadvantages in terms of outcomes and complications as a non-pharmacologic tool. Improvements in left ventricular ejection fraction and signs and symptoms of cardiogenic shock criteria were determinants. Nevertheless, complications during the implementation and use of the device were established; in this manner, the evaluation and treatment of each patient separately are imperative. Consequently, more studies on this relevant topic are needed.

## Introduction and background

Cardiogenic shock (CS) is one of the most common complications and the leading cause of death in patients with a diagnosis of cardiac ischemia. The left coronary artery that supplies most of the left ventricle is the most frequently affected artery, which leads to left heart failure and subsequently right heart failure. The pump's exhaustion due to cardiac insults provokes the left ventricular diastolic and systolic pressures to compensate improperly. As a result, there is low circulation and oxygen supply to the cardiac cells, affecting the stroke volume. This increases the venous pressure backward and overwhelms the pulmonary circulation. In addition to these events, the cardiac output is insufficient while supplying blood, oxygen, and nutrients to the tissues, which leads to end-organ hypoperfusion, systemic vasoconstriction, and generalized hypoxia [1,2].

CS criteria are defined by systolic blood pressure (SBP) of <90 mmHg for more than 30 minutes or supportive intervention to preserve the systolic pressure when it is >90 mmHg, in addition to the evidence of organ failure (mental impairment, urine output of <30 ml/hour, or cold extremities) [3]. The frequency of CS is 5%-10% in acute cardiac ischemia and has increased twofold between 2004 and 2014. The mortality associated with this condition is around 50% in patients with acute myocardial infarction (AMI) [1,4,5].

Currently, coronary revascularization and the use of inotropes are the principal keys to approaching AMI associated with CS [1]. Although clinical and interventional treatment is a tool for this scenario and is established as part of the guidelines, the management is sometimes not enough to meet the needs of a patient with AMI complicated with CS. For that reason, some devices can be used in this situation, such as the intra-aortic balloon pump (IABP), the left atrial-femoral artery bypass (TandemHeart, LivaNova, Houston, TX), the veno-arterial extracorporeal membrane oxygenation (VA-ECMO), and the Impella (Abiomed, Danvers, MA) device [6].

The Impella device is a tool that is increasingly used to provide mechanical support when there is heart failure provoked by an AMI [7]. This device consists of a microaxial pump that crosses the aortic valve toward the left cavity of the ventricle. It continuously extracts blood from the left cavity and unloads it into the arterial circulation, improving circulation, preventing systemic hypotension and tissue hypofunction, and decreasing the severity of the complications. However, some factors, such as age and lactate levels, could be decisive in their use [8,9].

**Figure 1:**
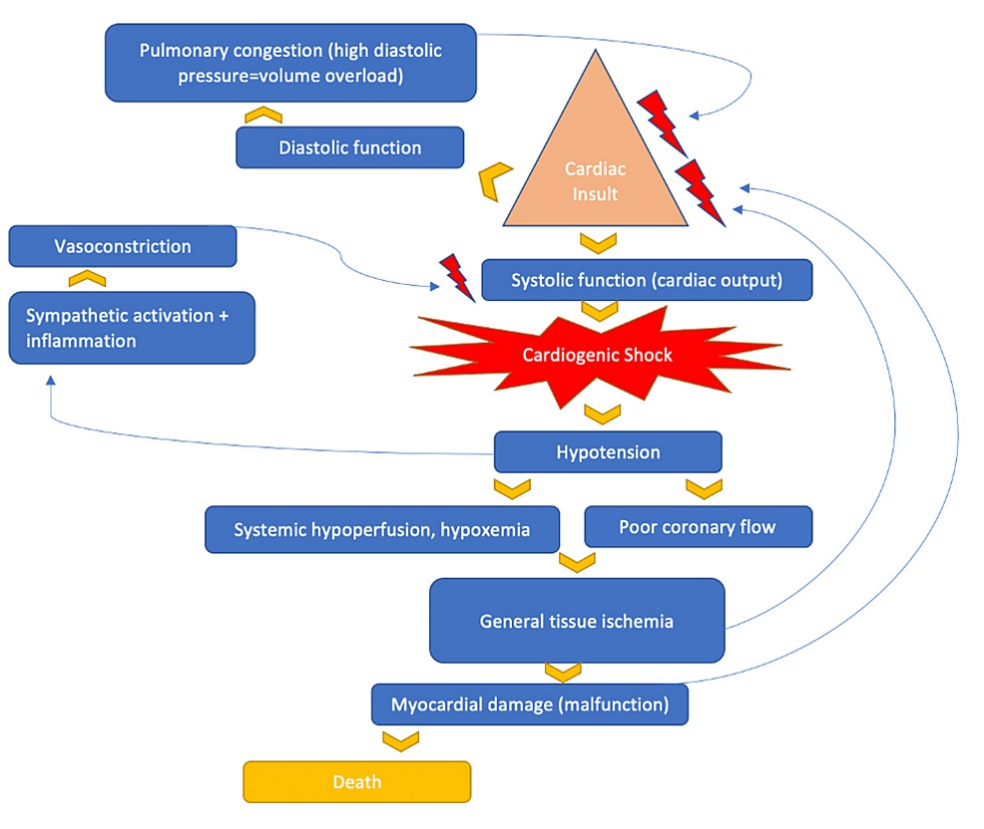
Pathophysiology of cardiogenic shock

Nowadays, the use of these appliances in the heart scenarios mentioned above has increased. The aim of interventional cardiology has rapidly and favorably changed. Even though some studies on the outcomes of using Impella devices in CS already exist, science must constantly update the information and integrate these devices into management to gain a better understanding of the effects in serious conditions such as CS. Given this, there are currently more research studies being conducted regarding this topic, and as evidence emerges, new mechanisms or protocols can be modified in order to deeply understand their uses in cardiology. In this systematic review, we seek to analyze various study results from the application of the Impella device to collect information on cardiovascular disease. Figure 1 illustrates the pathophysiology of CS.

Methods

We conducted a systematic review following the Preferred Reporting Items for Systematic Reviews and Meta-Analyses (PRISMA) 2020 checklist [10]. As secondary data from published articles were used, ethical approval was not taken into consideration.

Search strategy

PubMed, ScienceDirect, and Google Scholar were used as databases to find relevant studies. We selected articles published in the last five years. We applied the Medical Subject Heading (MeSH) search strategy to obtain related articles and also keywords to find the appropriate information for "Impella Device in Cardiogenic Shock."

Keywords such as Impella device, cardiogenic shock, and heart assist device were used in conjunction with the MeSH strategy. Furthermore, Booleans such as "AND" and "OR" were utilized to connect the different terms mentioned above. Table 1 is a summary of the search strategy for this systematic review.

**Table 1:** Bibliographic search strategy detailed and results yielded

Database	Search strategy	Filters	Results
PubMed	Cardiogenic shock OR ("Shock, Cardiogenic/complications" {Majr} OR "Shock, Cardiogenic/mortality" {Majr} OR "Shock, Cardiogenic/physiology" {Majr} OR "Shock, Cardiogenic/therapy" {Majr}) AND Impella device OR Heart assist device OR Left Ventricular Assist Device OR ("Heart-Assist Devices/mortality" {Majr} OR "Heart-Assist Devices/therapeutic use" {Majr} OR "Heart-Assist Devices/therapy" {Majr})	Age group (65+ years), 2017-2022, all location, all settings, humans, all type of studies (free full text), and English language	523 articles
Google Scholar	Allintitle: Impella Device in Cardiogenic Shock	2017-2022 and all article types	22 articles
ScienceDirect	Impella Device in Cardiogenic Shock	2017-2022; article type: research articles; subject area: medicine and dentistry	334 articles

Inclusion criteria

We have included articles written in English in the last five years and peer-reviewed, studies done in humans, and all types of studies and settings. We focused this research on the intervention of the Impella device in cardiogenic shock in patients who are 65+ years old. 

Exclusion criteria

We did not include articles written in languages other than English, studies done in animals or in patients younger than 65 years old, gray literature, unpublished articles, or articles done before 2017. Population, intervention, comparison, and outcomes (PICO) criteria were used as a framework for our eligibility criteria.

Data selection and extraction

Two researchers decided to select and extract studies related to the topic. The researchers resolved this systematic review step by step and discussed different study designs and outcomes and the inclusion and exclusion criteria for screening and assortment. If there was an inconclusive article, both would analyze it and take a decision after asking the third and fourth author. 

Through the three databases, 877 articles were selected after the removal of duplicates by EndNote (Clarivate, Philadelphia, PA) software. We applied the inclusion and exclusion criteria with the search strategy at the beginning of the screening and identified articles directly related to the topic. The researchers analyzed the articles during the screening process, and a total of 30 articles were selected.

Quality assessment

The studies selected by two independent researchers were qualified to decrease the probability of bias and to ensure proper selection and high-quality scores. Clinical trials were evaluated using the Cochrane risk of bias assessment tool (RoB 2). Observational studies (case-control and cohort) were assessed by the Newcastle-Ottawa scale, case report/series by Case Report (CARE) guideline checklist, and systematic review/meta-analysis by Assessing the Methodological Quality of Systematic (AMSTAR) checklist. Every paper was reviewed using the previously mentioned quality assessment tools, and those with an average quality score of more than 75% were selected.

Results

**Figure 2:**
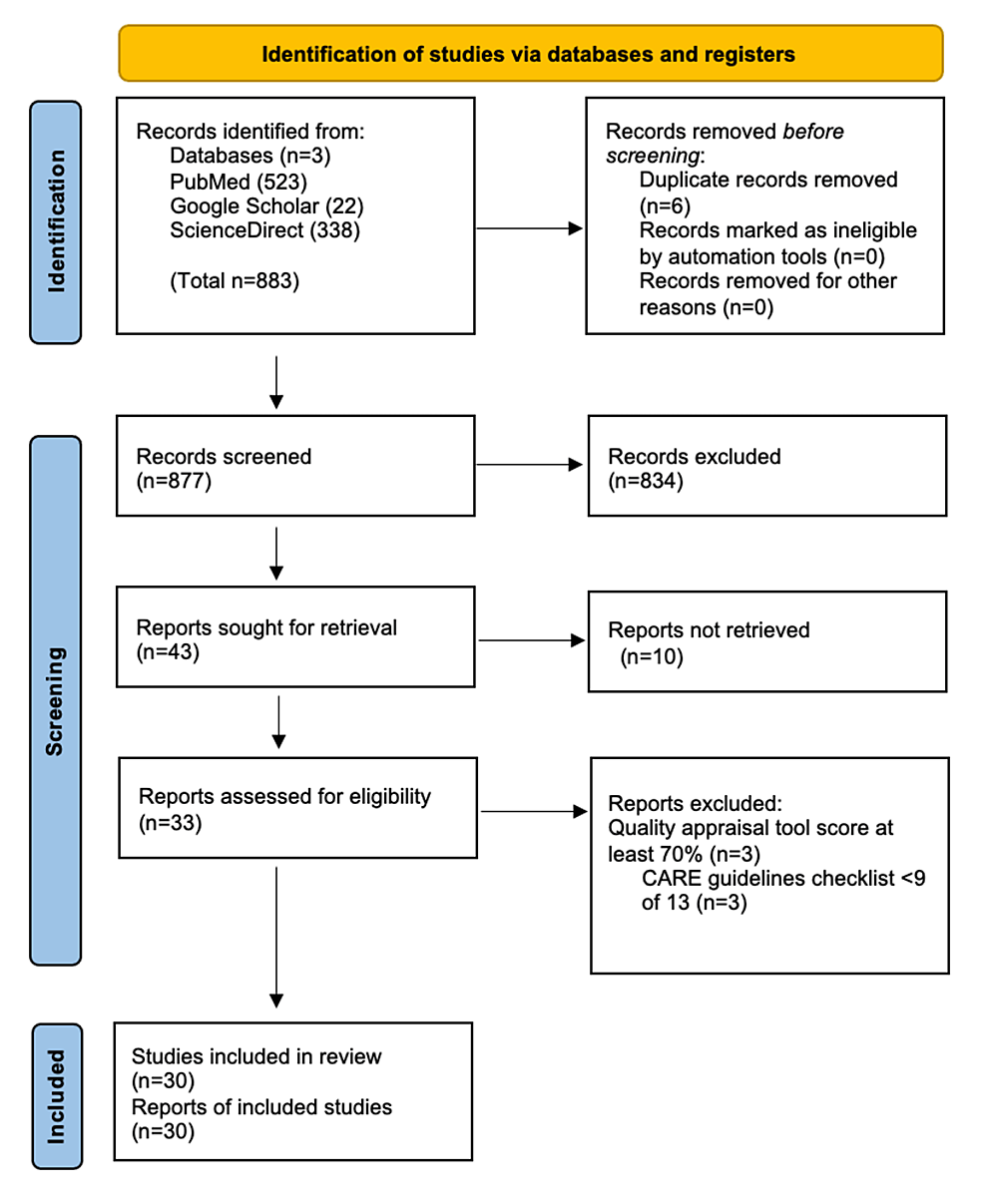
Flow diagram of study selection CARE: Case Report

Using different methods across the databases, we found 877 records in total. Three hundred thirty-eight of them were found in ScienceDirect, 523 in PubMed, and 22 in Google Scholar. Before the screening and selection of the studies, we manually removed six duplicates. The 877 articles were thoroughly assessed for relevance in their titles and abstracts. As a result, we were able to exclude 834 articles due to their lack of relevance regarding the topic, aims, and inclusion and exclusion criteria. The PRISMA guidelines were followed, and they are exhibited in Figure 2 [10].

Therefore, 43 articles were sought for retrieval, and after going through all of them, we further removed 10 studies. We assessed 33 articles for eligibility, and three articles did not achieve the quality appraisal required and thus were excluded. Table 2 [6,8,9,11-37] contains the details of the relevant 30 articles included.

**Table 2:** Impella device articles extracted and evaluated CS: cardiogenic shock; VA-ECMO: veno-arterial extracorporeal membrane oxygenation

	Author and year	Type of study	Patient age	Purpose of the study	Results	Conclusion
1	Abdullah et al., 2021 [9]	Cohort	69.0±18.0 years	The evaluation of results and prognosticators in a real-world scenario by the Impella device in CS.	At baseline, lactate levels were 4.7 mmol/L, and the mortality rate was 81%. Age and lactate were related to mortality.	Age and lactate help in the selection for Impella placement because mortality is high in sick CS patients.
2	Afana et al., 2021 [6]	Case series	29-69 years, mean of 55.5 years	Transcaval access route as an alternative for peripheral Impella application in CS.	Due to the challenging peripheral entry, 10 patients underwent the Impella 5.0 device placement procedure successfully in CS by transcaval access.	Transcaval access was a successful method for the insertion of emergency mechanical cardiac support when peripheral artery access was inadequate.
3	Ashraf et al., 2020 [11]	Case report	70 years	A patient with mitral regurgitation and an acute myocardial infarction (AMI) was treated with percutaneous coronary intervention, a stent, and Impella support.	After experiencing Impella support, percutaneous coronary intervention, and a stent placement for acute mitral regurgitation associated with an AMI, the patient regained hemodynamic stability and showed clinical improvement at one-month follow-up.	The Impella Cardiac Power Pump associated with MitraClip (Abbott Laboratories, Chicago, IL) repair was successful and secure in this patient.
4	Attisano et al., 2020 [12]	Case report	70 years	Report on a case management with a Takotsubo syndrome diagnosis complicated by severe mitral regurgitation.	A patient with left ventricular outflow tract obstruction and severe mitral regurgitation assisted by the Impella device was discharged after one week in stable conditions.	The Impella device is suggested as being an appropriate implement for patient recovery.
5	Bochaton et al., 2020 [8]	Prospective randomized trial	60.3±12.3 years	Twelve patients available out of fifteen with acute myocardial infarction and CS treated with inotropes and intra-aortic balloon pumps (IABPs) were evaluated besides adding the Impella device.	The cardiac power index showed no substantial difference between 12 and 96 hours. In both groups, the left ventricular ejection fraction was similar at baseline and after one month. The rate of bleeding was high and common.	In patients with an AMI and CS, treatment with vasopressors, an intra-aortic balloon pump, and a combination with Impella did not improve left ventricular ejection fraction during the initial phase of treatment or one month later. Combining them all together could be harmful.
6	Burzotta et al., 2019 [13]	Cohort	72±10 years	A report on Impella device placement as a protective tool for highly risky patients.	At 12 months, patient mortality was 10.5%. In these, left ventricular ejection fraction improved by ≥35% compared to 22.1% before Impella-protected percutaneous coronary intervention.	In complicated and high-risk patients, the Impella device placement was maintained for protected percutaneous coronary intervention. Improved left ventricular ejection fraction outcomes and advanced percutaneous coronary intervention were associated.
7	Dhruva et al., 2021 [14]	Cross-sectional	Mean of 65.4 years	Observe trends in the application of mechanical cardiac support devices in patients treated for AMI with CS with percutaneous intervention.	Intravascular microaxial left ventricular assist device usage increased (4.1%-9.8%), while IABP usage decreased (34.8%-30.0%).	In this study, a 2.5-fold increase in intravascular microaxial eft ventricular assist devices and a decrease in IABP placement were seen as a trend.
8	Doshi et al., 2018 [15]	Cohort	Male: 61.1±13.1; female: 67.3±15.1	Investigate patient gender variances in the setting of CS in short-term survival and in-hospital outcomes after experiencing percutaneous coronary intervention with the Impella association.	In terms of survival after 30 days, there was no difference between the gender groups. Additionally, in both groups, the outcomes were improved by starting Impella prior to a percutaneous coronary intervention.	Gender differences were not observed to be different, and mechanical cardiac assistance may improve survival and reduce problems in both sexes.
9	Elder et al., 2018 [16]	Case series	50±22 years	A report on patients with acute pulmonary embolism (PE) in shock with proof of hemodynamic instability undergoing the Impella device.	There were five cases of pulmonary embolism associated with shock. The cardiac index increased from a mean of 1.69 L/minute/m^2^ to 2.5 L/minute/m^2^ in 24 hours. No other complications were found.	The Impella device demonstrated hemodynamic improvement, correcting pulmonary embolism-induced shock, and increasing patient survival.
10	Ellert et al., 2018 [17]	Case report	65 years	A report on a case of biventricular Impella (BiPella) support in a patient with refractory out-of-hospital cardiac arrest.	Impella showed improvement in right ventricle function, whereas the left ventricle had no changes. Twelve months later, the patient was in good condition.	A patient who had refractory out-of-hospital cardiac arrest was successfully treated with biventricular Impella support.
11	Flierl et al., 2017 [18]	Case series	68±11 years	Twenty-one patients maintained by the Impella device (17 patients with CS) were investigated for acquired von Willebrand syndrome.	The presence of acquired von Willebrand syndrome was found in 95% of the patients.	A frequent concern when using the Impella device to support the left ventricle is acquired von Willebrand syndrome. It could be the cause of bleeding problems in patients at high risk.
12	Goldfarb et al., 2018 [19]	Case series	57.0±12.7 years	A report on patients assessed to demonstrate coagulation problems (von Willebrand syndrome) related to mechanical cardiac support.	Von Willebrand syndrome workup was recommended for 18 patients with mucosal bleeding (50%) and a drop in hemoglobin level (50%), respectively. The type II von Willebrand qualitative defect is consistent with all reported cases of high or normal low-molecular-weight multimers.	Temporary mechanical cardiac support devices were associated with the loss of high-molecular-weight multimers. When receiving short-term mechanical circulatory support, these findings may have implications for any future interventions or heart transplants.
13	Hamanaka et al., 2020 [20]	Case report	78 years	A case report on change in coronary microcirculation during mechanical cardiac support in a patient with ST-elevation myocardial infarction.	The Impella device increased coronary flow and decreased the index of microcirculatory resistance through the use of PressureWire (Abbott Vascular, Santa Clara, CA). Myocardium was found to be exempt of acute damage by cardiac magnetic resonance.	This case indicates that the Impella device improves coronary microcirculation by unloading the left ventricle, lowering endocardial pressure, and subsequently lowering coronary microcirculation resistance.
14	Hasegawa et at., 2021 [21]	Case report	66 years	A case report on a patient with AMI, CS, electric storm, and difficult peripheral access who was treated with Impella via the left axillary, the removal of the IABP, and ventricular pacing.	The Impella device combined with atrial overdrive pacing with difficult peripheral access, CS, and electric storm was successful.	Axillary artery access for the Impella device was effective, and atrial overdrive pacing was successful in the treatment of a patient with CS and electric storm with difficult aorto-iliac access.
15	Hritani et al., 2019 [22]	Cohort	Median of 74 years	The evaluation of outcomes with Impella device placement at 30 days in the United States.	Forty-four patients were evaluated; 27 were in high-risk percutaneous coronary intervention, and 13 were in CS. CS mortality in 30 days was 76.9% and in the high-risk percutaneous coronary intervention 11.1%. Renal dysfunction was the most common complication.	Despite the theoretical use of the Impella device, the outcome in CS continues to be poor.
16	Iannaccone et al., 2021 [23]	Meta-analyses	Median age of 61.9 years	Outcomes of hemodynamic support with the Impella device.	Seventeen studies were analyzed with 3933 patients. CS related to myocardial infarction was 79.6%. The 30-day mortality was 47.8%. Based on meta-regression analysis, the Impella 5.0 and Impella Cardiac Power were related to a higher survival rate.	Prior percutaneous coronary intervention with no cardiac arrest using the Impella device was recommended with better outcomes, but at 30 days, Impella-treated mortality in CS is still high.
17	Karatolios et al., 2019 [24]	Case series	Mean age of 78.3±9.6 years	Explore the capability and outcomes of Impella 2.5 in patients diagnosed with severe aortic stenosis and CS who experienced percutaneous aortic valvuloplasty with or without percutaneous coronary intervention.	Four patients (50%) died, and the most common cause was sepsis by pneumonia. The remaining patients completed hospital discharge and remained well at 30-day follow-up after hospitalization.	The Impella 2.5 assistance in tolerating percutaneous coronary intervention in extremely risky patients can be used to help hemodynamically assisted patients with severe aortic stenosis and CS after percutaneous balloon aortic valvuloplasty.
18	Karatolios et al., 2021 [25]	Cohort	68.96±11.56 (Impella) and 61.25±10.40 (VA-ECMO)	Compare outcomes in patients with CS assisted by Impella or VA-ECMO.	In this analysis, 423 patients were analyzed; 300 patients were supported by Impella and 123 by VA-ECMO. No difference was found in survival rates. Hemorrhage and leg ischemia occurred more often by VA-ECMO.	The hospitalized and six-month survival rates for patients who received Impella or VA-ECMO treatment were similar. In VA-ECMO than in Impella, vascular complications were more common.
19	Kuchibhotla et al., 2017 [26]	Cohort	59.6±6.3 years	Explore the clinical utility of percutaneous BiPella in CS complicated by biventricular failure.	Right ventricular Impella was given to 20 patients. Fifty percent of people died. Most complications involved bleeding and hemolysis. Better results were seen in younger patients.	BiPella supports cardiac function and reduces cardiac pressure during all causes of CS.
20	Lazkani et al., 2017 [27]	Cohort	Early Impella: 69 years (62-77); late Impella: 66 (58-73)	Aim to know in-hospital all-cause mortality and other complications and one-year mortality.	In this study, there were 262 patients included. As a result, 181 on early Impella had major adverse cardiac and cerebral events of 17.1% compared to late Impella at 59.3%. In-hospital morality on early Impella was 8.8% compared to 48.1% on late Impella. One-year mortality was 21.5% for early versus 53.1% for late.	Positive outcomes both in-hospital and after one year were associated with prompt application of the Impella device.
21	Maniuc et al., 2019 [28]	Case-control	Mean age of 61±13	Study the use of the Impella device in patients with non-ischemic CS compared with ischemic CS.	Twenty-five patients with non-ischemic CS were compared to 50 patients with ischemic CS. No difference was established in both groups at 30-day survival and in-hospital mortality. As a result, showing similarities in terms of treatment use.	In both ischemic and non-ischemic CS, temporary Impella device therapy has been proposed as a bridge to recovery.
22	Markus et al., 2020 [29]	Case series	67.81±14.18 years	The evaluation of left ventricular assistance with the Impella device on the renal resistive index by Doppler ultrasound in CS.	The Impella device was reduced considerably from 0.66±0.08 to 0.62±0.06 (P<0.001) during placement in 15 patients with no effect on blood pressure.	The Impella device in CS reduces renal resistive index without affecting blood pressure, enhancing renal perfusion to support renal organ protection.
23	Meraj et al., 2017 [30]	Case series	69.8±14.2 years	Assess post-procedural outcomes in patients with AMI and CS prior to or after percutaneous coronary intervention.	Pre-percutaneous coronary intervention (20 patients) (55%) had better survival at discharge versus post-percutaneous coronary intervention (16 patients) (18.8%). The Kaplan-Meier curve presented reduced survival in post-percutaneous coronary intervention of 48.1% compared to pre-percutaneous coronary intervention of 12.5%.	Pre-percutaneous coronary intervention with the Impella 2.5 device demonstrated better survival compared to post-percutaneous coronary intervention. Additionally, patients who had Impella placement following percutaneous coronary intervention appeared to have a poor prognosis at 30 days.
24	Nakamura et al., 2019 [31]	Case report	70 years	Management in a patient with AMI supported with Impella device converted to left ventricular assist device as bridge to recovery.	In a patient with a valvular disorder complicated by ischemia, the Impella and paracorporeal left ventricular assist devices led to the recovery of cardiac activity and posterior removal from mechanical cardiac support.	Mechanical cardiac support initiation was shown to successfully bridge to recovery in a patient with valvular disorder complicated by ischemia.
25	Nishikawa et al., 2021 [32]	Case report	Mean of 57.75	A report on four patients with Takotsubo syndrome complicated by CS resuscitated with the Impella device.	Three out of four patients who presented with an increase in left ventricular end-diastolic pressure were helped by the Impella device support during resuscitation.	In a patient with Takotsubo syndrome complicated by CS, the Impella device support contributed to an improvement in the cardiac index.
26	Oezkur et al., 2021 [33]	Cohort	63 years (54-70)	The evaluation of laboratory numbers could help determine the risk of hemorrhage complications.	Thirty-two (53.3%) patients showed major or fatal bleeding complications, despite heparin use to maintain normal activated partial thromboplastin time. In this study, bleeding was associated with acquired von Willebrand syndrome.	Acquired von Willebrand syndrome was associated with bleeding complications, and activated partial thromboplastin time monitoring was unsatisfactory to prevent these events. A patient with an Impella device should have their anticoagulation better assessed.
27	Pappalardo et al., 2017 [34]	Case-control	53 years (46–65)	Compare the outcomes of patients with CS who received VA-ECMO and Impella to those who received only VA-ECMO.	Patients with VA-ECMO and Impella had an inferior hospital mortality rate of 47% versus 80% for VA-ECMO only. The road to recovery was higher with additional therapy (68% versus 28%) compared with VA-ECMO.	Compared to VA-ECMO alone, VA-ECMO combined with Impella may improve outcomes in patients with CS.
28	Pieri et al., 2018 [35]	Cohort	Impella: 66.3±10.7; IABP: 65.2±11.7	Evaluate in-hospital survivance and the level of recovery after six months of mechanical cardiac support placement (IABP or Impella device) in patients with AMI complicated with CS and planned percutaneous coronary intervention.	The Impella group compared to the IABP group exhibited lower myocardial injury by measuring troponin peak of 3831 ng/dl (1241-8436) versus 16581 (7802-23675) and also creatine-phosphokinase peak of 893 UI/L (584-4082) versus 5797 (2483-9292). The change of left ventricular ejection fraction at six months was greater than 45% (38-52) versus 40% (33-45), respectively.	Impella may minimize cardiac muscle damage and improve recovery in AMI with CS prior to percutaneous coronary intervention, increasing left ventricular ejection fraction at six months.
29	Tarabichi et al., 2020 [36]	Case series	Mean age of 61.2±10.7	Define the function of the axillary Impella devices in the setting of CS.	During hospitalization, 21 patients out of 40 (52%) died. Seven patients presented complications such as right extremity ischemia or neuropathy (three patients) and a nonfunctional Impella device needing substitution (four patients).	Patients with CS who need short-term cardiac mechanical support may find success with axillary Impella therapy.
30	Udesen et al., 2019 [37]	Randomized controlled trial	68 years (59-76)	Check the hypothesis that left cardiac mechanical support with the Impella increases survival in AMI associated with CS.	In this analysis, 314 patients were screened, and 100 patients were randomized. Patients showed a median lactate of 5.5 mmol/L (range: 3.7-8.8 mmol/L), median systolic blood pressure of 76 mmHg (range: 70-80 mmHg), and median left ventricular ejection fraction of 20% (range: 10%-30%).	The DanGer Shock trial will be the first randomized trial to assess if left cardiac mechanical support with Impella can improve survivance in AMI complicated by CS.

## Review

Discussion

The effect of the Impella device on patients with CS from various etiologies is covered in this section. This includes results, timing, route accessibility, and difficulties. A diagnosis of CS is based on SBP <90 mmHg for more than 30 minutes, supportive therapy to maintain SBP above 90 mmHg, and signs of end-organ damage (altered mental status, decreased urine output, or cold extremities) [3]. Numerous articles published in recent years have shown that the use of the Impella device on patients experiencing cardiogenic shock leads to better results. The studies evaluated in this systematic review showed multiple aspects and outcomes regarding the use of Impella devices. The well-established benefits revealed the improvement of cardiac function and systemic circulation and decreased the severity of the illnesses.

Outcomes

In studies conducted on patients with CS treated with the Impella device, gender was initially the first variable examined, and after the analysis of the results, it was determined that there was no difference between males and females in terms of the outcomes [15]. The improvement in cardiac function was perceived after using the Impella device in these patients. However, before this device was implanted, risk factors, such as age and lactate levels, had to be considered before deciding the management [9].

Different scenarios, including mitral valve regurgitation (MVR), Takotsubo syndrome, pulmonary embolism (PE), out-of-hospital cardiac arrest, severe aortic stenosis, valvular disorders associated with ischemia, biventricular failure, ischemic and non-ischemic CS, acute myocardial infarction (AMI), and percutaneous coronary intervention in high-risk patients, could be complicated with CS, but the benefits of this device were higher in all circumstances [11-13,16,17,26,28,31,32,35]. These studies also demonstrated the efficacy of cardiac mechanical support by the Impella device, improving survival and becoming an important tool to manage CS despite the etiology. During hospitalization decision-making by physicians regarding cardiac mechanical support, there was a 2.5-fold increase in the placement of microaxial left ventricular assisting devices (4.1%-9.8%) compared to a reduction in IABP (34.8%-30.0%) between 2015 and 2017. Consequently, it is a significant trend nowadays [14].

The Impella device has been demonstrated to enhance coronary microcirculation by emptying the left ventricular chamber, which contributes to a decrease in endocardial pressure and, as a result, lower coronary circulation resistance, resulting in favorable filling within the microcirculation. During coronary angiography, PressureWire was used to evaluate this finding; later, cardiac magnetic resonance imaging demonstrated no myocardial damage, confirming this statement [20]. Pieri et al. compared patients treated with the Impella device to IABP, measuring cardiac enzymes. The Impella device showed an inferior peak of troponins and creatine phosphokinase compared to IABP; furthermore, at six months, the left ventricular ejection fraction was 45% and 50%, respectively, proving minor muscle injury and the recovery of left ventricular ejection fraction. All the data was statistically significant [35]. In an analysis presented by Markus et al., patients with AMI associated with CS who had the Impella device implanted exhibited a decreased renal resistive index, improving the perfusion of the kidney through the renal arteries without changing the blood pressure and maintaining appropriate circulation. The performance of a Doppler ultrasound in renal circulation corroborated the results [29]. Pappalardo et al. showed that the Impella device in association with other devices as VA-ECMO was significant, showing a superior recovery and reducing hospital mortality by 47% compared to 80% for VA-ECMO alone [34].

Timing

The factor "time" must be deliberately selected. In a comprehensive review and meta-analysis, Impella was used as a tool before percutaneous coronary intervention, demonstrating no cardiac arrest and having a positive impact on survival rate. Nevertheless, at 30 days, the mortality rate for CS patients remained high [23]. In a study conducted in Arizona, 262 high-risk patients had the implantation of Impella devices, which were later analyzed. The main finding was a significant difference in the outcomes related to "time." They came to the conclusion that "prompt positioning" seemed to have better results than "belated positioning" in terms of in-hospitalization and one-year all-cause death rates [27].

In another article, Meraj et al. discussed that prior to percutaneous coronary intervention, the implantation of the Impella 2.5 had an improvement in survival compared to post-percutaneous coronary intervention, shown by the Kaplan-Meier curve at 30 days (12.5% versus 48.8%) [30].

Route of access

Peripheral access can be challenging in a hospital setting, especially in patients with the diagnosis of CS. Therefore, it is important to mention that in this instance, the use of transcaval emergency mechanical cardiac support Impella 5.0 implantation was successful [6]. Axillary artery access has also shown effectiveness in patients with an aorto-iliac approach [21]. Another analysis was made when the axillary Impella was implanted in patients who needed temporary cardiac support, demonstrating better results [36]. Hence, in these cases, such routes of access can be feasible to consider.

Complications

Bleeding was the most common adverse effect during the use of Impella devices [8]. Oezkur et al. found that 53.3% of the patients had coagulation issues, specifically related to acquired von Willebrand syndrome, despite being treated with heparin to maintain a normal activated partial thromboplastin time range. In this situation, the anticoagulation factor should be considered in patients with Impella placement and should be monitored during its use [33]. The phenomenon of acquired von Willebrand disease is common; thus, assessing high-risk patients is a crucial component to prevent bleeding as a complication [18]. According to Goldfarb et al., the loss of high-molecular-weight multimers is a key factor in the diagnosis of acquired von Willebrand syndrome. Blood tests to identify von Willebrand factor activity, fibrinogen, factor VIII, or von Willebrand factor antigen were required in 10 cases where bleeding (mucosal hemorrhage or low hemoglobin) was detected. These patients with these findings also showed high, normal, or elevated levels of "low-molecular-weight multimers." Due to these findings, type II von Willebrand syndrome (qualitative deficiency) has been recognized. As a result, future intraoperative placement of similar devices, heart transplants, or operations while receiving mechanical cardiac support could all have implications [19]. In a study conducted in a tertiary care facility, renal dysfunction was found to be a common adverse effect in patients with cardiogenic shock and high-risk percutaneous coronary intervention. In another one, hemolysis was also reported as a side effect of the application of biventricular Impella (BiPella) in patients with biventricular failure [22,26]. According to Tarabichi et al., seven out of 21 patients experienced complications such as arm ischemia (axillary access), neuropathy, or Impella device malfunction [36].

Twelve patients with AMI and CS were included in a prospective randomized trial; unfavorable outcomes were also presented and reported; these findings were related to the placement of an Impella in combination with IABP and inotropes, showing no significant changes in left ventricular end-diastolic pressure at one month and being deemed "harmful" by bleeding complications [8]. Despite the theory of hemodynamic improvement using this device, the use of Impella device in CS remains poor in some cases [22]. All of these findings and outcomes may be crucial in the development of new devices and new strategies for the treatment of patients with CS.

Given those mentioned above, in the majority of the studies, the Impella device has been shown to improve the outcomes in patients with CS caused by various etiologies. Nevertheless, it is essential to consider each patient separately because each of them has unique characteristics that may have an impact on the final result, inside and outside of the hospital. When it comes to timing, complications, and access routes, it is indeed critical to learn from errors and use new techniques if problems appear during the treatment. It is also important to develop similar tools such as in the DanGer Shock trial, which was demonstrated to be the first designed powered randomized trial, which determined that mechanical cardiac support with Impella device positioning can increase the expectancy of life in patients suffering from AMI associated with CS [37]. In this manner, we could address and refine patient decisions with advanced criteria depending on the case.

Limitations

Our systematic review revealed some limitations. First, only English-language studies from three databases between 2017 and 2022 were included in this study. Second, different study types, such as observational, randomized clinical trials, systematic reviews, and meta-analyses, were selected; as a result, there are variations in the samples and statistical methods, making our revision susceptible to bias. Third, due to the high complexity of the disease and the use of mechanical cardiac support, some studies were conducted with small samples, and finally, the total follow-up was variable in different studies. In this scenario, it is important to highlight that studies with larger samples are desirable to get more conclusive results in order to assure the presence of significant benefits among these patients and later on have a better understanding of the complications.

## Conclusions

We concluded, based on the information collected, that most patients with a diagnosis of CS have an improvement using the Impella device. This evaluation was founded on the left ventricular ejection fraction, improvement in the CS criteria signs and symptoms, and favorable response in the follow-ups. There are feasible alternatives by other access routes with the same benefit when patients present difficult access during device implantation by a conventional route. The Impella device may also improve patient outcomes when it comes to timing, a crucial consideration in decision-making. Although any patient could experience an adverse event, it is essential to evaluate and treat each patient distinctively based on their characteristics. We corroborated that the use of this device in these kinds of patients would be widely useful during cardiac support to improve their management and prognosis. Future recommendations include leading and piloting more studies, especially cohorts and randomized controlled trials with larger samples, combinations of therapies, longer follow-up times, and monitoring of complications. These ideas are mentioned in order to gain a clear view of the results after employing this device on CS and perhaps fill some gaps and discrepancies found during this systematic review. Therefore, it is important to mention that more investigation regarding the Impella device is needed to refine and elaborate future evidence.
